# Activation and Inhibition of TMEM16A Calcium-Activated Chloride Channels

**DOI:** 10.1371/journal.pone.0086734

**Published:** 2014-01-29

**Authors:** Yu-Li Ni, Ai-Seon Kuan, Tsung-Yu Chen

**Affiliations:** Department of Neurology, Center for Neuroscience, University of California Davis, Davis, California, United States of America; Monell Chemical Senses Center, United States of America

## Abstract

Calcium-activated chloride channels (CaCC) encoded by family members of transmembrane proteins of unknown function 16 (TMEM16) have recently been intensely studied for functional properties as well as their physiological roles as chloride channels in various tissues. One technical hurdle in studying these channels is the well-known channel rundown that frequently impairs the precision of electrophysiological measurements for the channels. Using experimental protocols that employ fast-solution exchange, we circumvented the problem of channel rundown by normalizing the Ca^2+^-induced current to the maximally-activated current obtained within a time period in which the channel rundown was negligible. We characterized the activation of the TMEM16A-encoded CaCC (also called ANO1) by Ca^2+^, Sr^2+^, and Ba^2+^, and discovered that Mg^2+^ competes with Ca^2+^ in binding to the divalent-cation binding site without activating the channel. We also studied the permeability of the ANO1 pore for various anions and found that the anion occupancy in the pore–as revealed by the permeability ratios of these anions–appeared to be inversely correlated with the apparent affinity of the ANO1 inhibition by niflumic acid (NFA). On the other hand, the NFA inhibition was neither affected by the degree of the channel activation nor influenced by the types of divalent cations used for the channel activation. These results suggest that the NFA inhibition of ANO1 is likely mediated by altering the pore function but not through changing the channel gating. Our study provides a precise characterization of ANO1 and documents factors that can affect divalent cation activation and NFA inhibition of ANO1.

## Introduction

Calcium-activated chloride (Cl^−^) channels (CaCCs) play important physiological roles, such as regulating trans-epithelial transport, controlling smooth muscle contractility, amplifying odorant signals in olfactory receptor neurons, and modulating action potentials in hippocampal neurons [1], [2], [3]. Recently, members of the family of transmembrane proteins with unknown function 16 (TMEM16) were identified, and experiments from multiple groups indicated that the gene products encoded by TMEM16A and TMEM16B formed CaCCs [4], [5], [6]. Another member of the TMEM16 family, TMEM16F, was shown recently to form a small conductance calcium-activated cation channel [7], while other evidence suggested that TMEM16F may also function as phospholipid scramblase or different types of Cl^−^ channels [8]. These TMEM16 family members are thought to form dimeric molecules [9], via a homotypic dimerization domain located at the N-terminal cytoplamic region of the proteins [10].

The CaCC channel molecules encoded by TMEM16A and TMEM16B are also called anoctamin 1 (ANO1) and anoctomin 2 (ANO2), respectively [6]. ANO1 is thought to be the major CaCC in epithelial cells, while ANO2 modulates the action potential of hippocampal neurons [11] and controls the sensory signal transduction in olfactory receptor neurons [12], [13], [14], [15], [16], [17]. The activation and inhibition properties of these channels had been studied before the channel cloning [15], [18], [19], [20]. It has been shown that CaCCs open in response to sub-micromolar/micromolar concentrations of free Ca^2+^. The pore of the channel is “lyotropic” [21], [22]–anions with a larger molecular size have a permeability ratio larger than that of Cl^−^ (namely, P_X_/P_Cl_ >1, where X is an anion). CaCCs can be reversibly inhibited by inhibitors/blockers such as niflumic acid (NFA) [15], [19], [23]. For the last 20 years, these functional properties were used as hallmarks to search for the genuine CaCCs [3], [22]. The ANO1 channel appears to show these functional characteristics described in many early studies of the CaCCs in epithelial cells [3], [24], [25].

While CaCCs consist of well-defined functional properties, a technical complication is frequently encountered in studying these channels–the “rundown” or “desensitization” of CaCCs. The rundown of CaCCs could affect the apparent affinity of Ca^2+^ activation, the degree of current rectification, or even the calculated permeability ratios of various anions. From the literature it can be found that the apparent affinity of Ca^2+^ from dose-dependent activation curves of CaCCs varies significantly, and this large variation cannot be fully explained by alternative splicing of the TMEM16A protein [26]. For example, at −60 mV the Ca^2+^ sensitivities among various alternatively spliced variants of TMEM16A differed by ∼4–6 fold [26]. At the same voltage, the reported half-effective concentration of Ca^2+^ (K_1/2,Ca_) in the literature ranged from <100 nM [27] to >2 µM [6], [28]. It is not known if channel rundown or other experimental factors contributed to the widely varied apparent affinities of CaCCs reported in the literature.

In this study we employed a fast solution exchange method to induce ANO1 current upon switching the intracellular solution from a zero-Ca^2+^ solution to a solution containing specified Ca^2+^ concentrations ([Ca^2+^]). We circumvented the problem of channel rundown by normalizing the Ca^2+^-induced current to the maximally-activated current obtained within a time period in which the channel rundown is negligible. We found that two other divalent cations, Sr^2+^ and Ba^2+^, can activate ANO1 to the same level as that activated by the saturating [Ca^2+^]. On the other hand, Mg^2+^ cannot induce ANO1 current, but it appears that Mg^2+^ can bind to the divalent cation binding site(s) to antagonize channel activation by Ca^2+^. We also discovered that the apparent affinity of a well-known CaCC blocker, NFA, was inversely related to the occupancy of anions in the pore of ANO1. On the other hand, the NFA block was not affected by the degree of channel activation. The mechanism of the NFA block of ANO1 is not well understood. Our experiments revealed functional properties that may help to unravel the mechanism for the NFA inhibition on the ANO1 channel.

## Results

### Channel Rundown Does Not Alter the Sensitivity of ANO1 to Ca^2+^

**Figure 1:**
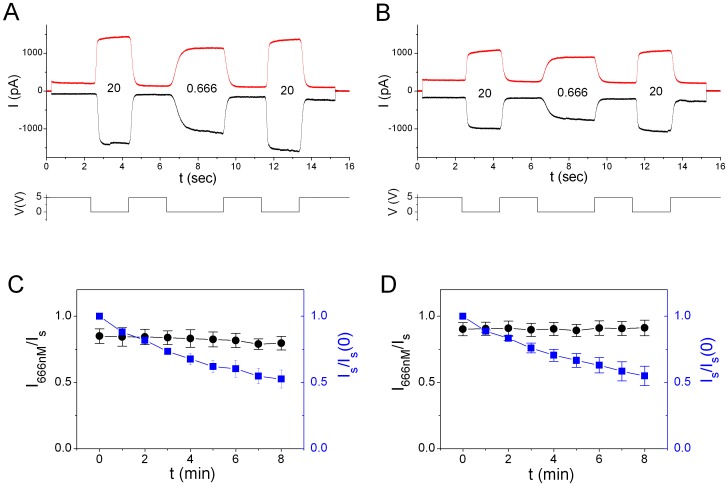
Current run-down does not affect the sensitivity of ANO1 to Ca^2+^-activation. (A) Original recording traces of Ca^2+^-induced ANO1 current at −40 mV (black) and +40 mV (red). The experiments were initiated by clamping the voltage to ±40 mV followed by applying the indicated free [Ca^2+^] (in µM) to the intracellular side of the membrane patch. The change of the 5-V digital signal shown at the bottom was recorded simultaneously to indicate the time when the fast-solution exchange pipes were switched. (B) ANO1 current from the same patch as in A recorded 8 minutes later. (C & D) Sensitivity of ANO1 to Ca^2+^ activation is not changed throughout the recording. Experiments like those shown in A & B were conducted once per minute at −40 mV (C) and +40 mV (D). The saturated current (I_s_) thus obtained was normalized to the I_s_ obtained at t = 0. The decline of the I_s_/I_s_(0) ratio (blue symbols) indicates the rundown of the ANO1 current. Black symbols are the ratio of the currents induced by 0.666 µM and 20 µM Ca^2+^ of the same trace (N = 4).

CaCCs are notoriously known to undergo prominent channel rundown, a poorly understood phenomenon that frequently complicates the experiments. We first examined whether the channel sensitivity to Ca^2+^ activation was changed in the process of rundown. Fig. 1 A shows the ANO1 current induced by 0.666 µM [Ca^2+^] (I_666 nM_) bracketed by maximal currents (I_s_) induced by 20 µM [Ca^2+^], a saturating [Ca^2+^]. A repeated trace recorded 8 minutes later is shown in Fig. 1 B. In both cases, the two I_s_’s within the same recording sweep did not show a significant difference, but the amplitude of I_s_ was reduced between sweeps separated by minutes because of the current rundown. Fig. 1 C & D illustrate, at −40 mV and +40 mV respectively, the time course of current rundown by normalizing I_s_ of each sweep to that of the initial current sweep (I_s_(0)). The ratio I_666 nM_/I_s_ from each sweep was also calculated as a measure of the sensitivity of ANO1 to Ca^2+^ activation. The results showed that ANO1 revealed a prominent current rundown, with ∼50% current attenuation after 8 minutes. However, the sensitivity of ANO1 to Ca^2+^ activation remained the same throughout the 8-min recording period as indicated by the unchanged ratio of I_666 nM_/I_s_ (Fig. 1 C & D).

### Sensitivities of ANO1 Activation by Different Divalent Cations

**Figure 2:**
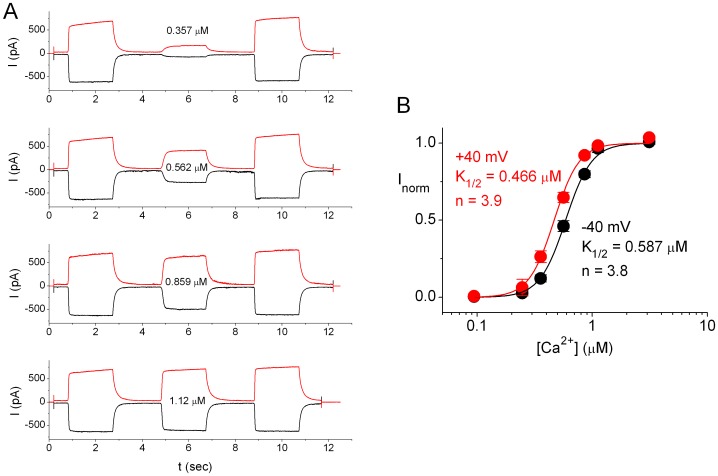
Concentration-dependent activation of ANO1 by Ca^2+^. (A) Original recording traces show the current of ANO1 activated by the application of various free [Ca^2+^] (in µM) bracketed by the applications of saturating [Ca^2+^] (20 µM). Black traces: −40 mV; Red traces: +40 mV. (B) Dose-response curves of ANO1 activation by Ca^2+^ at −40 mV (black) and +40 mV (red). Data were derived from recordings like those shown in A. Data were averaged from 2–8 patches. Peak currents activated by different free [Ca^2+^] were normalized to those of the saturated current in the same recording trace, and the normalized values were used to construct the dose-response curves. Solid curves were the best fit to eq. 1, with indicated values of K_1/2′_s and Hill coefficients.

**Figure 3:**
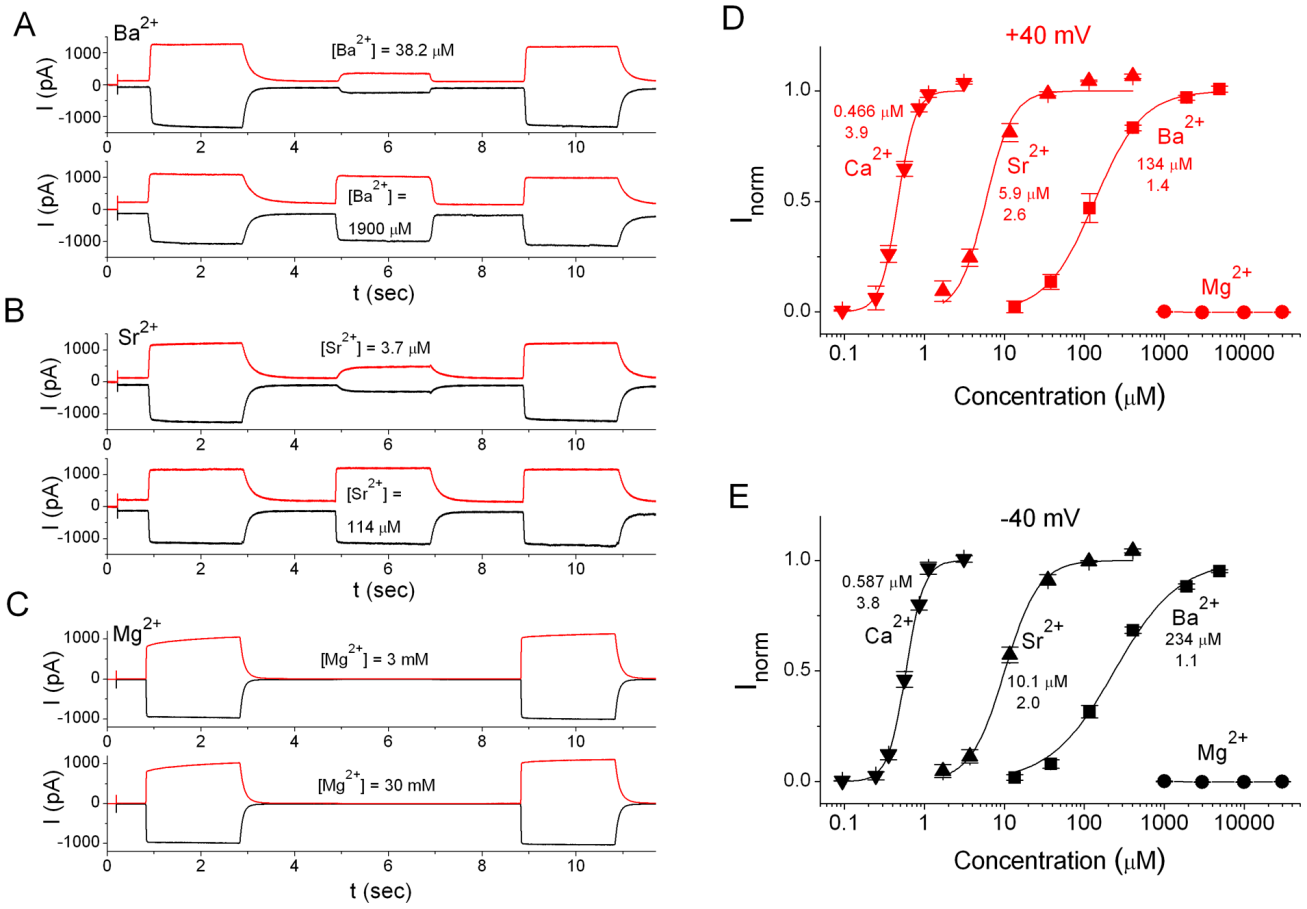
Comparison of the sensitivities of ANO1 activation by different divalent cations. (A, B & C). Original recording traces showing the activation of ANO1 by various concentrations of divalent cations at −40 mV (black) and +40 mV (red). The saturated currents before and after the experimental concentrations of divalent cations were induced by 20 µM free Ca^2+^. (D & E) Dose-dependent activations of ANO1 by various divalent cations. Solid curves were the best fit of data points to eq. 1, with values of K_1/2′_s (in µM) and Hill coefficients shown. The calcium-activation curves are the same as those shown in Fig. 2 B. N = 4 and 5 for the Sr^2+^ and the Ba^2+^ experiments, respectively. Data points with Mg^2+^ (N = 4) are connected by straight line segments. Membrane voltages are indicated on top of each panel.

As shown in Fig. 1 A & B, the current rundown of ANO1 within a recording sweep of <20 sec was negligible. We therefore employed the same method shown in Fig. 1 to test the sensitivities of ANO1 to different divalent cations. We first examined the Ca^2+^ dose-dependent activation of ANO1 (Fig. 2 A) by normalizing the currents activated by various [Ca^2+^] to I_s_ induced by 20 µM free [Ca^2+^]. The averaged dose-response curves of Ca^2+^ activation at +40 and −40 mV are plotted in Fig. 2 B. The mean of the data from different patches were used to fit to a Hill equation (eq. 1), with half-activation [Ca^2+^] (K_1/2,Ca_) of 466 nM and 588 nM at +40 mV and −40 mV, respectively, and a Hill coefficient (n_Ca_) of ∼4. We further performed the experiments using Ba^2+^ (Fig. 3 A), Sr^2+^ (Fig. 3 B), or Mg^2+^ (Fig. 3 C) as the activation ligand. In these experiments, the I_s_ in each recording sweep was also induced by 20 µM [Ca^2+^]. It can be seen that saturating [Ba^2+^] and [Sr^2+^] can activate the same maximal current as the I_s_ induced by saturating [Ca^2+^]. In comparison with the current activated by Ca^2+^, the Sr^2+^- and Ba^2+^-activated current appeared to disappear more quickly when the divalent cations were removed. This is likely due to a faster dissociation rate of Sr^2+^ and Ba^2+^ from the channel upon washout. Indeed, the dose-response curves of Sr^2+^ and Ba^2+^ revealed lower apparent affinities than the apparent Ca^2+^ affinities. Curve fitting showed that K_1/2,Sr_ = 5–10 µM and n_Sr_ = 2–2.6, while K_1/2,Ba_ = 130–230 µM and n_Ba_ = 1.1–1.4 (Fig. 3 D & E). On the other hand, no ANO1 activation was detected by using up to 30 mM [Mg^2+^] (Fig. 3 C).

### Mg^2+^ Appears to be a Competitive Inhibitor of Ca^2+^ in ANO1 Activation

**Figure 4:**
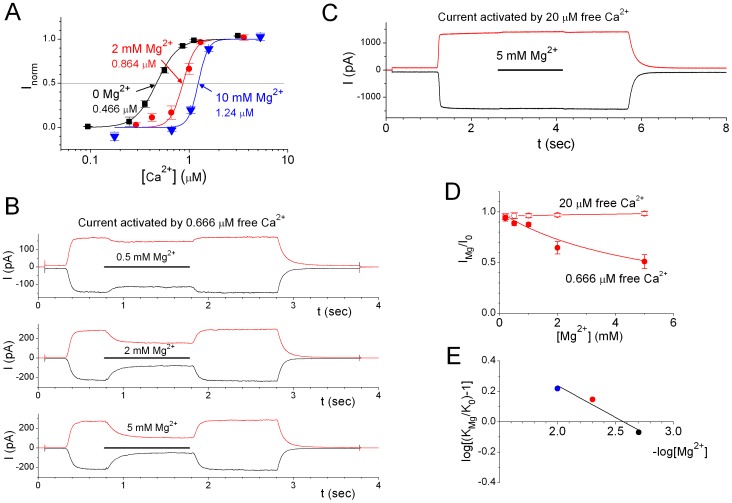
Mg^2+^ shifts the Ca^2+^-activation curve of ANO1. (A) Dose-response curves of ANO1 activation by Ca^2+^ in the absence (black) or in the presence of 2 mM (red; N = 4) and 10 mM (blue; N = 6) intracellular Mg^2+^. The values of K_1/2,Ca_ in each condition are shown. (B) Mg^2+^ inhibits ANO1 current induced by non-saturating [Ca^2+^] in a dose-dependent manner. (C) ANO1 current activated by saturating [Ca^2+^] was not inhibited by 5 mM Mg^2+^. (D) Comparison of the dose-dependent Mg^2+^ inhibitions of ANO1 currents induced by 0.666 µM and 20 µM free Ca^2+^. Data were obtained from experiments like those shown in B in 3 (0.666 µM) and 5 patches (20 µM). Data points of 20 µM Ca^2+^ are connected by straight line segments while those of 0.666 uM Ca^2+^ are fitted to eq. 2 with an apparent half-inhibition concentration of 5.4 mM. (E) Schild plot of the K_1/2′_s of the Ca^2+^-activation curve of ANO1 in the absence and presence of Mg^2+^. K_1/2′_s of the Ca^2+^-activation curves shown in A were determined, and those obtained in 2 (black), 5 (red) and 10 mM (blue) of Mg^2+^ (K_Mg_) together with that in the absence of Mg^2+^ (K_0_) were used to construct the Schild plot. The straight line fitted to the data points crossed the horizontal axis at pMg = ∼2.6, indicating that ∼2.5 mM [Mg^2+^] reduces the ANO1 affinity for Ca^2+^ by half.

Intracellular Mg^2+^ was unable to activate ANO1 current, yet, it reduced the channel sensitivity to the activating divalent cations. Fig. 4 A shows that 2 mM (colored in red) or 10 mM (colored in blue) [Mg^2+^] in the intracellular solution shifts the Ca^2+^ dose-response curve to the right. The inhibition of the Ca^2+^-induced current by Mg^2+^ is shown in the experiments depicted in Fig. 4 B, in which the solutions with and without Mg^2+^ contained the same total [Ca^2+^] of 0.910 mM and total [EGTA] of 1 mM. Without Mg^2+^ in the solution, the calculated free [Ca^2+^] was ∼666 nM. Adding Mg^2+^ in this solution at the same pH should increase the free [Ca^2+^] even though Mg^2+^ binds to EGTA only weakly (Table S1). Yet, the switch of the solution from the zero-Mg^2+^ solution to Mg^2+^-containing solutions resulted in an inhibition of the current. This Mg^2+^ inhibition, however, was not observed in the experiment using saturating [Ca^2+^] (Fig. 4 C), suggesting that Mg^2+^ may act as a competitive inhibitor of Ca^2+^. We thus examined Mg^2+^ inhibition in two ways. In Fig. 4 D, [Mg^2+^]-dependent inhibitions were carried out when 20 µM or 0.666 µM free [Ca^2+^] was used to induce the ANO1 current. In Fig. 4 E, a Schild plot was constructed using the values of K_1/2,Ca_ estimated from the dose-response curves like those shown in Fig. 4 A. It can be seen from the Schild plot that the K_1/2,Ca_ was increased 2-fold by ∼2.5 mM [Mg^2+^], a physiologically relevant [Mg^2+^].

### Anion Permeabilities of ANO1 Under Bi-ionic Conditions

**Figure 5:**
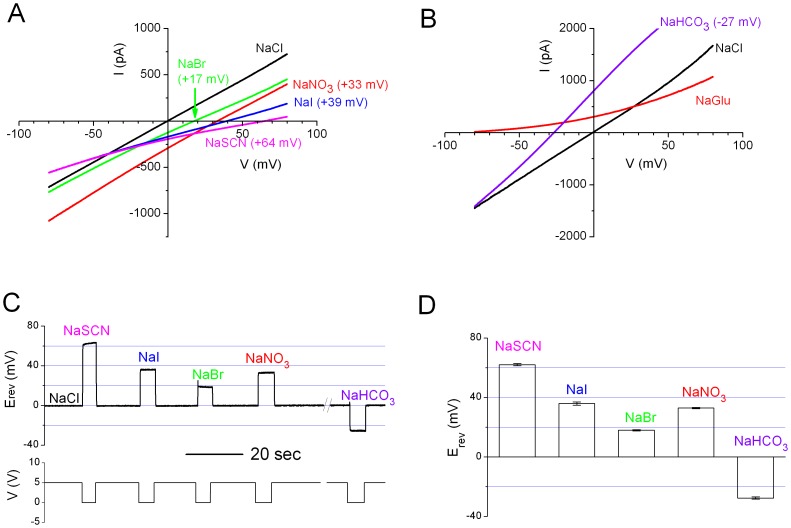
E_rev_ measurements of Ca^2+^-activated ANO1 currents in various anion solutions. (A) I–V curves of ANO1 with 140 mM extracellular NaCl and 140 mM sodium salts of various anions. All currents were induced by a ramp protocol from −80 mV to +80 mV. [Ca^2+^] = 20 µM. All recordings were from the same patch. Voltages in parentheses are the zero current potential in each anionic condition. (B) I-V curves of ANO1 with 140 mM extracellular NaCl and two different intracellular ionic conditions. For the trace labeled with NaGlu (which denotes Na-glutamate), 140 mM NaGlu was used. For the trace labeled with NaHCO_3_, 10 mM NaCl and 130 mM NaHCO_3_ were present in the intracellular solution. (C) Measuring bi-ionic potentials in different intracellular anions using I = 0 current clamp recordings. [Ca^2+^] = 20 µM throughout the recording. Pipette solution contains 140 mM NaCl. The intracellular solution initially contained 140 mM NaCl, and was changed to various salt solutions as indicated by the 5-V digital signal used to trigger the movement of solution delivering pipes. The salt concentrations were the same as those described in A & B. (D) Bi-ionic potentials averaged from experiments like those shown in A, B & C (N = 8).

To characterize the functional properties of ANO1 more extensively, we studied the anion permeability of the channel pore. This was accomplished by measuring the reversal potential (E_rev_) of the channel current under bi-ionic conditions. Two different approaches were employed to determine the E_rev_. First, the E_rev_ of I-V curves from voltage-clamp recordings were measured (Fig. 5 A & B). Alternatively, we employed current clamp (I = 0) recordings and monitored the voltage (which represented the E_rev_ of the membrane current) when the intracellular Cl^−^ was replaced by other anions (Fig. 5 C). The E_rev_ measured by these two different approaches were nearly identical to each other, so the results were averaged together (Fig. 5 D). Overall, with SCN^−^, I^−^, NO_3_^−^, or Br^−^ as the intracellular anion, E_rev_ was positive (E_rev_ = 62.0±2.4 mV, 35.9±3.3 mV, 32.9±1.1 mV, and 18.0±1.2 mV, respectively, for these four anions without junction potential correction). After junction potential correction, the calculated permeability ratios (P_X_/P_Cl_) were ∼11, ∼4.1, ∼3.5, and ∼2.1 for SCN^−^, I^−^, NO_3_^−^, or Br^−^, respectively. On the other hand, E_rev_ = −27.6±2.4 mV (P_HCO3_/P_Cl_ = ∼0.3 after junction potential correction) when HCO_3_ **^−^** was the major intracellular anion (Fig. 5 D), while E_rev_ was more negative than −80 mV when glutamate was the intracellular anion (Fig. 5 B). It should be noticed that [HCO_3_ **^−^**] was less precise than the concentrations of other anions. However, given that the permeability of HCO_3_ **^−^** is much smaller than that of Cl^−^, it is safely concluded that the permeability ratios for these anions followed the sequence of SCN^−^>I^−^>NO_3_ **^−^**>Br^−^>Cl^−^>HCO_3_ **^−^**>Glutamate. These results are consistent with the lyotropic property of the CaCC pore [18], [22].

**Figure 6:**
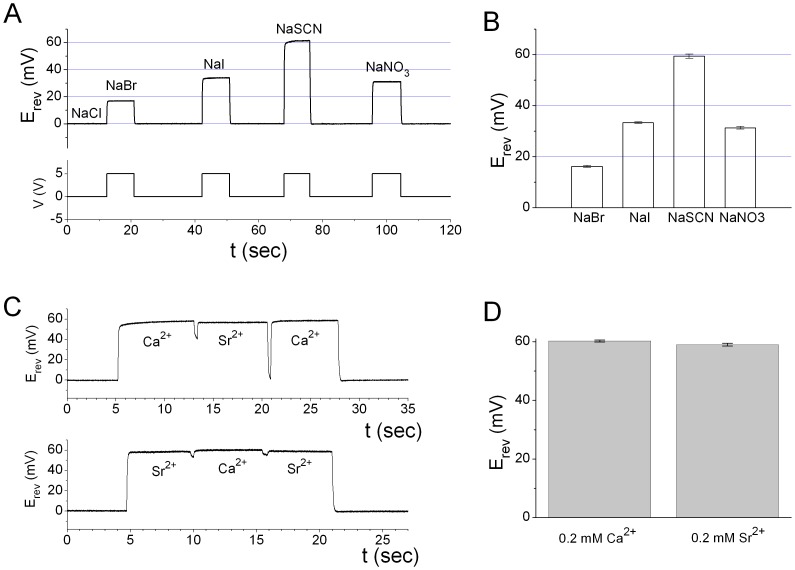
E_rev_ of the Sr^2+^-activated ANO1 current with various intracellular anions. (A) I = 0 current clamp experiments similar to that shown in Fig. 5 C. (B) Averaged bi-ionic potentials in various intracellular anion solutions from the experiments like that shown in A (N = 4) (C & D) Comparison of the E_rev_’s of Ca^2+^- and Sr^2+^-induced currents in 140 mM intracellular NaSCN. Extracellular solution contained 140 mM NaCl. Values shown in D were the average of 4 different patches.

Because ANO1 can be activated by divalent cations other than Ca^2+^, we also examined the permeation properties of the channel activated by 20 µM Sr^2+^, using the current-clamp (I = 0) recording approach under bi-ionic conditions (Fig. 6). The Sr^2+^-activated current has slightly smaller E_rev_ in comparison with the results shown in Fig. 5 C, but the permeability sequence of various anions in the presence of Sr^2+^ was the same as that in Ca^2+^. In one set of experiments (Fig. 6 B), E_rev_’s were 59.4±0.8 mV, 33.4±0.3 mV, 31.3±0.5 mV, and 16.2±0.4 mV with intracellular SCN^−^, I^−^, NO_3_ **^−^**, and Br^−^, respectively, without correcting for the junction potential (N = 6). Because the measurement of E_rev_ could be imprecise if the amplitude of the current varies from patch to patch, we further compared the E_rev_’s of the Ca^2+^-activated and the Sr^2+^-activated current from the same patch, using SCN^−^ as the intracellular anion (Fig. 6 C). The averaged voltage shift induced by SCN^−^ in Sr^2+^ is only ∼1 mV smaller than that in Ca^2+^ (Fig. 6 D). Although this difference is statistically significant using pair-t test (n = 4), the 1-mV difference in E_rev_ results in a very small difference of the calculated P_SCN_/P_Cl_ ratio between the Ca^2+^- and the Sr^2+^-activated channels. These results indicated that the ANO1 pore opened by Ca^2+^ and Sr^2+^ have very similar anion permeability properties.

### NFA Block of ANO1 is Affected by the Occupancy of Permeant Ions in the Pore but not by the Degree of Channel Activation

**Figure 7:**
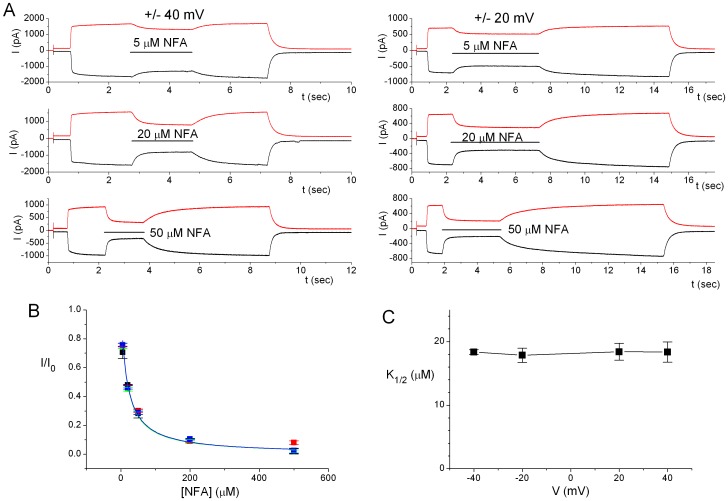
Niflumic acid (NFA) block of ANO1 is not voltage dependent. (A) Original recording traces of ANO1 blocked by various [NFA] at ±40 mV (left three panels) and ±20 mV (right three panels). Horizontal lines indicate the application of NFA. (B) Dose-response inhibition of ANO1 by NFA at different membrane voltages. Colored squares depicting data points from four different voltages (black: −40 mV; red: +40 mV; green: −20 mV; blue: +20 mV) are nearly superimposed. N = 6–11. Solid curves (same colors as those of data points) were the best fit to eq. 2 with K_1/2_ values shown in C. (C) K_1/2_ of NFA inhibition of ANO1 is not voltage dependent. The fitted values of K_1/2_ from B are plotted as a function of voltage.

NFA is a known inhibitor of CaCCs, but the mechanism of the NFA inhibition of CaCC remains elusive [19], [23]. We conducted NFA inhibition experiments by directly applying NFA to the intracellular side of the excised inside-out patch using a fast solution exchanger. Fig. 7 A shows examples of recording traces for the NFA inhibition on the ANO1 current induced by 20 µM Ca^2+^ at ±40 mV (left three panels) or at ±20 mV (right three panels). The averaged dose-dependent NFA inhibitions at these four different voltages are plotted in Fig. 7 B. The half-blocking concentrations (K_1/2_) of NFA were in the range of 18–20 µM and were not voltage dependent between −40 mV and +40 mV (Fig. 7 C).

**Figure 8:**
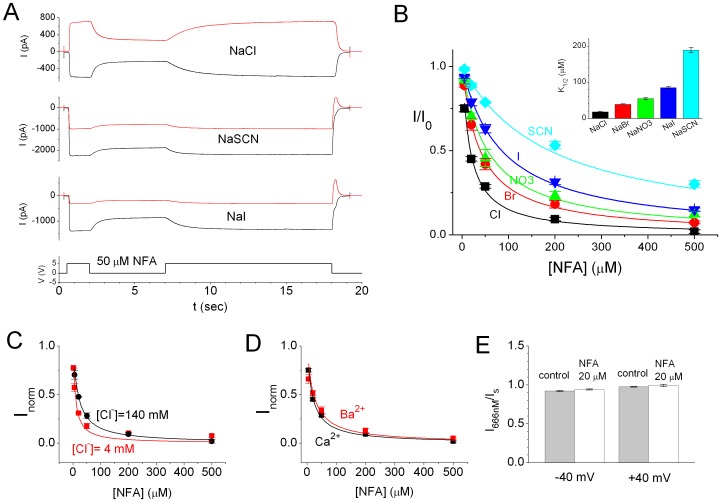
NFA block of ANO1 is affected by the occupancy of permeant ions in the pore but not by the degree of channel activation. (A) Blocking ANO1 current with 50 µM NFA in different intracellular anions. All intracellular anion concentrations were 140 mM. V_m_ = ±20 mV. The 5-V digital signal at the bottom panel indicates the time of the switch of perfusion pipes. (B) Concentration-dependent NFA blockage of ANO1 in different intracellular anions. Inset shows the fitted K_1/2_ of the block in different intracellular anions. Data obtained in the Cl^−^ solution were averaged from 9–11 patches, while the rest were obtained from 3–5 patches. (C) Comparison of the NFA dose-response inhibition curves obtained in symmetrical [Cl^−^] of 140 mM (black) and 4 mM (red) at −40 mV. For the latter solution, Na-Glutamate (136 mM) was added to replace the reduced [NaCl]. The two regression curves in 140 mM NaCl (K_1/2_ = 18.4 µM) and 4 mM NaCl (K_1/2_ = 7.9 µM) are statistically different from each other. Data were from 6 different patches. (D) Comparing NFA sensitivities of the ANO1 current activated by 20 µM [Ca^2+^] (K_1/2_ = 17.9 µM) and 404 µM [Ba^2+^] (K_1/2_ = 22.6 µM). V_m_ = −20 mV. N = 5–11. No statistical difference between the two curves. (E) Sensitivity of ANO1 to Ca^2+^ activation is not reduced by NFA. Percentage of the ANO1 current induced by 666 nM Ca^2+^ at ±40 mV was compared in the absence (solid bars) and in the presence (open bars) of 20 µM NFA (N = 4). Experiments were performed in the same way as those shown in Fig. 1. No statistically significant difference between the experiments with and without NFA (p>0.05, t test).

The potency of the NFA inhibition of ANO1 was affected by intracellular anions. Fig. 8 A shows experiments in which ANO1 inhibitions by 50 µM NFA at ±20 mV were conducted in 140 mM intracellular Cl^−^, I^−^, or SCN^−^ (all Na^+^ salts) while the pipette solution contained 140 mM [Cl^−^]. At +20 mV (red traces), an inward current was observed in the I^–^ or SCN^–^containing solution because the E_rev_ values in these two intracellular solutions were ∼+35 mV and ∼+60 mV, respectively (Fig. 5 D). Whether at +20 mV or −20 mV, the percentage of current inhibition was the largest with NaCl, followed by NaI and then NaSCN. Fig. 8 B shows the dose-response curves of the NFA inhibitions in five different anions. The K_1/2′_s of NFA in 140 mM NaCl, NaBr, NaNO_3_, NaI, and NaSCN were (in µM): 17.9±1.1, 39.2±1.1, 54.8±3.4, 85.5±3.6, and 189.4±7.4, respectively (Fig. 8 B, inset). It is interesting that the apparent affinities of the NFA block appear to inversely correlate with the permeability ratios of these anions–the higher the permeability ratio for an anion, the lower the apparent affinity of the NFA block in the solution containing this anion. These results suggested that anion occupancy in the pore may affect the apparent affinity of the NFA block. To test this idea, we conducted NFA-blocking experiments in low [Cl^−^] conditions. Fig. 8 C shows a comparison of the NFA dose-dependent inhibition curves in symmetrical solutions containing 140 mM versus 4 mM NaCl. The apparent affinity of NFA inhibition was increased by more than 2 fold in 4 mM [Cl^−^] compared with that in 140 mM [Cl^−^], adding supportive evidence that the NFA block of ANO1 may be reduced by occupancy of ions in the pore.

On the other hand, the degree of the channel activation or the types of divalent cations used for channel activation did not affect the NFA block. Fig. 8 D compares the NFA dose-dependent inhibition curves in different divalent cations: 20 µM [Ca^2+^] versus 404 µM [Ba^2+^]. A free [Ca^2+^] of 20 µM is a saturating concentration for ANO1 activation whereas 404 µM [Ba^2+^] only activates ∼70–80% of the maximal current (Fig. 3 D & E). However, the NFA dose-dependent inhibitions in these two conditions were nearly identical, suggesting that the NFA inhibition mechanism is not related to the gating of the channel. Consistent with this observation, the sensitivity of ANO1 to Ca^2+^ was not changed with or without NFA–whether in the presence or absence of 20 µM NFA, 666 nM [Ca^2+^] activated ∼90% of the saturated current (Fig. 8 E).

## Discussion

We have rigorously examined the activation, anion permeability, and NFA block of ANO1 in this study. Because research approaches and experimental conditions were different in previous studies, the reported values of K_1/2,Ca_ varied significantly from <100 nM to several µM in the literature [6], [27], [29], [30]. A prominent channel rundown of ANO1 could contribute to these variations. For example, if ANO1 is activated sequentially from low to high [Ca^2+^], the percentage of the current activation by low [Ca^2+^] will be over-estimated because the maximal current of the same patch is gradually reduced as the channel rundown proceeds. We suspect that the very low K_1/2,Ca_ value reported in some studies of ANO1 (for example, K_1/2,Ca_ = 63 nM at +80 mV [27]) may have this problem. On the other hand, if saturating [Ca^2+^] is first applied to the patch followed by the exposure of the channel to non-saturating [Ca^2+^], the degree of current activation by lower [Ca^2+^] will be under-estimated. Considering these possibilities, we employed an experimental protocol that the divalent cation-activated current was normalized to the saturated current obtained in the same recording sweep of <20 sec, a short time period in which the ANO1 rundown was negligible (Fig. 1 B).

### Activation and Inhibition of ANO1 by Divalent Cations

Using such an experimental protocol, we were able to construct reliable dose-response curves of the channel activation by various divalent cations. We found that Ca^2+^, Sr^2+^, and Ba^2+^ all activated the channel to the same maximal current level, although with different apparent affinities. The K_1/2_ of the ANO1 activations by Ca^2+^, Sr^2+^, and Ba^2+^ were ∼0.5 µM, ∼5 µM, and ∼100–200 µM, respectively (Fig. 3). The binding of divalent cations likely triggers an allosteric conformational change in ANO1, leading to channel opening. Accordingly, the apparent affinities of the channel activation by these divalent cations should not be considered as the true binding affinities of the binding site(s). Interestingly, the Hill coefficients of the dose-response activation curves for Ca^2+^, Sr^2+^, and Ba^2+^ correlated with K_1/2′_s in a way that the most potent ligand, Ca^2+^, had a largest Hill coefficient near 4 while the Hill coefficiencts for Sr^2+^ and Ba^2+^ activation were ∼2 and 1–1.5, respectively. The Hill coefficient of a dose-dependent curve was usually considered to represent the “cooperativity” of ligand bindings, which could involve the gating conformational change subsequent to ligand bindings [31]. Thus, it appears to be quite reasonable that a high apparent affinity of the divalent cation activation in ANO1 is associated with a large Hill coefficient in the dose-response curve.

In contrast to Ca^2+^, Sr^2+^, and Ba^2+^, Mg^2+^ is not effective in activating ANO1. This is not due to the failure of Mg^2+^ binding to ANO1. The fact that Mg^2+^ competitively inhibits the ANO1 current activated by Ca^2+^ indicated that Mg^2+^ binds to the same divalent cation binding site(s) in ANO1. This finding may be physiologically pertinent because K_1/2,Ca_ is affected by millimolar [Mg^2+^], a physiological relevant concentration. It should also be pointed out that many experiments in the literature included millimolar [Mg^2+^] in the intracellular solution, and these studies usually reported values of K_1/2,Ca_ in the µM range [29], [30], [32]. Our finding that Mg^2+^ competes with Ca^2+^ for binding to the activation sites could explain the higher values of K_1/2,Ca_ reported in these previous experiments.

The fact that Mg^2+^ cannot induce ANO1 current is probably due to a failure of coupling the Mg^2+^-binding to the opening of the channel. This situation is similar to the major divalent activation sites (with µM Ca^2+^ affinity) in the large conductance Ca^2+^-activated K^+^ channel (or BK channel) [33], [34], to which Sr^2+^ and Ba^2+^ can bind and activate the BK channel while Mg^2+^ binds and prevents the activation of the channel by other divalent cations [35]. The BK channel contains a separate set of divalent binding sites to which Mg^2+^ can bind and activate the channel [36], [37], [38]. The mechanism of Mg^2+^ activation of the BK channel was thought to be due to a modulation of the voltage-dependent BK channel activation [36], [37], [39]. In our experiments, we were unable to detect ANO1 current after applying up to 30 mM Mg^2+^ in the intracellular side. The recorded current in the absence of Ca^2+^ at ±40 mV was small and was not proportional to the amplitude of the saturated Ca^2+^-activated current (for example, compare recording traces in Fig. 1 A and Fig. 1 B). The current induced by saturating [Ca^2+^] also showed little rectification in the voltage range between −80 to +80 mV (Fig. 5). We therefore considered that ANO1 likely is a ligand-gated channel with little voltage-dependent activation, at least within the applied voltage range. Previous studies have shown a voltage-dependent activation of ANO1 current in low intracellular [Ca^2+^] upon jumping the membrane voltage from negative to positive potentials [5], [18], [40]. This is possible because of the difference of the K_1/2,Ca_ at different voltages. Although our results showed that the values of K_1/2,Ca_ at the voltages of +40 mV and −40 mV differed by only 100 nM, a large value of the Hill coefficient can lead to a significant increase of the channel activation when jumping the voltage from −40 mV to +40 mV at low [Ca^2+^].

### Anion Permeability and NFA Block in ANO1 Channel

The permeability of the CaCC pore for various anions has been studied before [18], [22]. We measured E_rev_ of the ANO1 current under bi-ionic conditions to examine the permeability ratio of various anions. Our results show nearly identical permeability ratios with those measured for the native CaCC from *Xenopus* oocytes in an early study [18], with a permeability sequence of P_SCN_ >P_I_ >P_NO3_>P_Br_ >P_Cl_ >P_HCO3_. Since ANO1 can be activated by other divalent cations, we wondered if the permeability ratio depends on the ligand that opens the channel. Our results show that ANO1 channels activated by Sr^2+^ and Ca^2+^ have E_rev_’s that differ by only 1–2 mV. In addition, the sequence of the permeability ratio was identical whether the channel was activated by Ca^2+^ or Sr^2+^, indicating that different activating ligands do not significantly alter permeation properties of the channel pore.

An interesting finding of the anion permeability of ANO1 is related to the ANO1 inhibition by NFA. A previous study had examined the inhibitions of native CaCC channels in the *Xenopus* oocyte membrane by NFA and several other blockers such as A9C, DPC, and DIDS [19]. This early study showed that inhibition of the CaCC of *Xenopus* oocytes by A9C, DPC, and DIDS were voltage dependent while the inhibition by NFA was not. The lack of voltage dependence of the NFA inhibition on ANO1 shown in our study is consistent with the finding in the CaCC of *Xenopus* oocytes. Because A9C and DPC mostly blocked outward current (inward Cl^−^ flux) and the half-activating concentrations of the intracellular blockers were much higher than those of the extracellular blockers, it was concluded that A9C and DPC blocked the *Xenopus* oocyte CaCC from the extracellular side. On the other hand, it was more difficult to determine the sideness of the NFA block in this early study because NFA blocked CaCC of *Xenopus* oocytes from intracellular and extracellular sides with similar affinities [19]. We attributed the inhibition of NFA within a few seconds shown in our study as an action mainly from the intracellular side because the inhibition of the channel was immediately observed with little delay upon the application of NFA in comparison with the delay for the current rise induced by Ca^2+^ application (Fig. S1). However, we also noticed that the rate of the current recovery upon NFA washout depended on the NFA concentration ([NFA]), suggesting that some NFA molecules may cross the membrane and inhibit the channel from the extracellular side or from the lipid-protein junction.

Regardless of the sideness of the NFA action, our results revealed that the apparent affinity of the NFA inhibition of ANO1 was inversely related to the permeability ratios of anions. Consistent with this finding, reducing [Cl^−^] also enhanced the affinity of NFA (Fig. 8 C). These results may suggest that the NFA inhibition of ANO1 is counteracted by the occupancy of anions in the pore. One possibility is that binding of NFA compresses the pore of the channel so that the anion flux is reduced. Occupancy of anions in the pore thus could reduce the inhibition of NFA by rendering the pore less collapsible. It is also possible that NFA binds to the intracellular pore region of ANO1 and the presence of anions in the pore competes off the binding of NFA. However, our experiments were unable to conclude whether the binding site for the NFA inhibition is located in the pore region. Conceivably, if the anion occupancy prevents the pore from collapse, the NFA binding site could be located at any place on the channel, including the protein-lipid junction given the non-polar nature of NFA. Although we were unable to reveal the NFA binding site, our experiments show that the type of divalent cations used to activate ANO1 or the degree of the channel activation does not affect the NFA inhibition (Fig. 8 D & E), suggesting that NFA inhibition may be mediated by altering the pore function but not by affecting the gating mechanism of the channel.

In summary, using protocols that minimize the impact of channel rundown, we have revealed several functional properties of ANO1 that have not yet been documented. We show that Ca^2+^, Sr^2+^, and Ba^2+^ activated ANO1 to the same maximal current level, but with different apparent affinities. Mg^2+^ was unable to activate ANO1 but this divalent cation appeared to compete with Ca^2+^ binding to prevent channel activation. We also demonstrate that the inhibition of ANO1 by NFA appeared to be antagonized by occupancy of anions in the pore. However, it will require further studies to determine whether NFA binds to the pore region of the channel.

## Materials and Methods

### Molecular Biology and Channel Expression

The mouse TMEM16A cDNA (NCBI reference sequence: NM_001242349.1) encoding ANO1 CaCC was a generous gift from Dr. Lily Jan. We subcloned the coding region of the cDNA into pEGFP-N3 vector (Clontech Laboratories, Inc.), resulting in a construct in which an enhanced green fluorescent protein (eGFP) was attached to the C-terminus of the ANO1 protein. The sequence was confirmed by commercially-available DNA sequencing service. This eGFP-attached ANO1 cDNA construct was used for channel expression throughout the study. The cDNA was transfected into human embryonic kidney (HEK) 293 cells using lipofectamine transfection methods [41], [42], [43]. A fluorescent light source and a GFP filter set (Chroma Technology Co.) in the DM IRB inverted microscope (Leica Microsystems) were used to identify transfected cells expressing channel proteins. Electrophysiological experiments were performed ∼24 to 72 hours after transfection.

### Solutions and Chemicals

All chemicals were reagent grade. Regular salts (such as NaCl, NaOH, CaCl_2_, SrCl_2_, BaCl_2_, MgCl_2_ or sodium salts of various anions) were purchased from Fisher Chemicals (Fair Lawn, NJ), J. T. Baker (Phillipsburg, NJ) or Sigma Chemicals Co. (St. Louis, MO). EGTA, HEPES, and NFA were from Sigma Chemicals Co. Excised inside-out patch-clamp recordings were performed throughout the study. The extracellular (pipette) solution in all experiments contained: 140 mM NaCl, 10 mM HEPES, 0.1 mM EGTA, pH = 7.4. The regular intracellular solution contained 10 mM HEPES and 140 mM sodium salt of Cl^−^ or other anions. Various concentrations of divalent cations were made by adding certain total concentrations of divalent cations (Cl^−^ salts) into the solution containing a defined concentration of EGTA. We defined the pipette solution as the “zero-Ca^2+^” solution. A solution containing a saturating [Ca^2+^] was made by adding 0.12 mM total [Ca^2+^] into the aforementioned zero-Ca^2+^ solution, resulting in a solution containing ∼20 µM free [Ca^2+^]. For making non-saturating [Ca^2+^] solutions, the solutions included 1 mM EGTA. To make solutions containing Sr^2+^ and Ba^2+^, only 0.1 mM EGTA was used. The pH of all solutions was adjusted to 7.4.

Because of weak Mg^2+^-EGTA binding, we referred the total [Mg^2+^] as the free [Mg^2+^]. The free [Ca^2+^], [Sr^2+^], and [Ba^2+^] were calculated using the MaxChelator program (Ver1.2) kindly provided by Chris Patton from the Stanford University. This program was developed based on the binding constants available in the National Institute of Standard and Technology (NIST) between EGTA and these three divalent cations. Table S1, Table S2, and Table S3 list respectively the calculated free [Ca^2+^], [Sr^2+^], and [Ba^2+^] used in this study. All added divalent cations were Cl^−^ salts. When the added salts resulted in an increase of [Cl^−^] larger than 2 mM, [Cl^−^] was adjusted by reducing the added amount of NaCl. For example, in a solution containing 5 mM, 10 mM or 30 mM MgCl_2_, only 130 mM, 120 mM or 80 mM of NaCl, respectively, were added to the solution.

### Electrophysiological Experiments

The patch-clamp recording pipettes were fabricated from borosilicate glass capillaries (World Precision Instruments) using PP830 electrode puller (Narishige International). When filled with the pipette solution, the electrode resistance was ∼1.5–3 MΩ. The experiments were carried out at room temperature (20–23°C) using the Axopatch 200B amplifier controlled by the Digidata I/O converter and the pClamp9 software (Axon Instruments/Molecular Devices).

Solution exchange was achieved using the SF-77 solution exchanger (Warner Instruments). After the excised inside-out patch mode was established, the tip of the patch pipette was positioned at the exit of the solution-delivery pipes of the SF-77 solution exchanger. The switch of solution pipes was controlled by a digital signal delivered from the Digidata 1320 digitizing board. The time required for the motor moving the solution-pipes to respond, the time for the solution junction passing the patch, and the time for the ligand to diffuse into the Ω-shaped inside-out patch added up to tens of ms (sometimes 100–200 ms) as the dead time before the ligand effect could be observed (Fig. S1). The recorded current is normally time-independent following fast solution exchange, but on infrequent occasions there is a slow increase of current. Therefore, we measured the current amplitude at the end of the solution exchange when the current amplitude reaches a steady-state maximal value. To start an experiment, the intracellular side of the patch was first perfused with the zero-Ca^2+^ solution. The membrane potential was then clamped to ±40 mV (or ±20 mV in some experiments) followed by a switch of the solution to a different solution containing certain free [Ca^2+^]. Fig. 1A illustrates a typical experiment in which the current was induced by Ca^2+^-containing solution three times: two with a solution containing the saturating [Ca^2+^] (20 µM free Ca^2+^) and one with a solution containing a specific [Ca^2+^] (0.666 µM). The current rundown within one recording sweep of <20 s was normally negligible (see the time course of the current rundown in Fig. 1 B & C). If the amplitudes of the saturated current recorded before and after the non-saturated current differed by >10% in the same recording sweep, the recording trace was discarded. To monitor the current rundown as shown in Fig. 1 A, the recording was repeated once per minute. The saturated current (I_s_) obtained in every recording sweep was normalized to the I_s_ of the initial recording sweep. To evaluate the sensitivity of the channel to divalent cations, the amplitude of the current induced by the activation ligand was measured and normalized to the averaged value of the two I_s_’s in the same sweep (see Fig. 1 C & D). The normalized value thus represents the percentage of the channel activation.

The anion permeability of the ANO1 pore was examined under bi-ionic conditions in which the extracellular solution contained 140 mM NaCl (the zero-Ca^2+^ solution). The intracellular solution contained 140 mM sodium salts of various anions with the exception of the HCO_3_ **^−^** experiment in which the solution contained 130 mM NaHCO_3_ and 10 mM NaCl. In all intracellular solutions for the bi-ionic experiments, 0.1 mM EGTA and 0.12 mM total Ca^2+^ or Sr^2+^ were added to the solution to activate the ANO1 channel (Free [Ca^2+^] or [Sr^2+^] were saturating concentrations). E_rev_ of the open channel was measured in two ways. First, under voltage-clamp configuration, a 3-sec voltage ramp from −80 mV to +80 mV was applied and the E_rev_ of the leak-subtracted I-V curve was then determined (see Fig. 5 A & B). The second method employed the current-clamp (I = 0) mode in which the patch was exposed to divalent cations throughout the recording (Fig. 5 C and Fig. 6 A & C). When the intracellular solution contained the same concentration of Cl^−^ as that in the extracellular side, the recorded voltage (E_I = 0_) was normally close to 0 mV. When the intracellular solution was replaced by the one containing other anions, the value of E_I = 0_ changed to a new value corresponding to the E_rev_ of the new ionic condition. It is important to point out that the measurement of E_rev_ can be imprecise if the amplitude of the ANO1 current is not within a proper range. Therefore, in permeability ratio measurements we only used the data obtained from patches with a maximal current in the range of 100 pA–1000 pA at +40 mV. All the presented E_rev_ values (and the recording traces for measuring E_rev_) are not corrected for junction potential difference between solutions because the junction potentials are relatively small. However, the reported permeability ratios were calculated based on the junction potential-corrected E_rev_. The correction for the junction potential results in very small changes in the calculated permeability ratios.

The inhibition of ANO1 by intracellular NFA was achieved by directly applying NFA to the intracellular side of the membrane patch using fast-solution exchange. Except where indicated, the ANO1 current was induced by saturating [Ca^2+^] followed by the application of various [NFA] in the solution containing the same [Ca^2+^]. The steady-state current (I) at the end of NFA application was divided by the current in the absence of NFA (I_0_) before and after the drug application to obtain the percentage of current remained in the presence of NFA.

### Data Analysis

Data analyses were conducted using the software of pClamp10 (Axon Instrument/Molecular Devices) and Origin 8 (OriginLab). All the averaged data were presented as mean ± S.E.M. For the analysis of ANO1 activation, the current induced by non-saturating concentrations of divalent cations were normalized to the maximally-activated current induced by 20 µM [Ca^2+^] in the same recording sweep. The percentage current activation (I_norm_) was plotted against the divalent cation concentration ([D]), and the dose-response activation curve was fitted to a Hill equation (eq. 1), I_norm_ = 1/{1+ (K_1/2,D_/[D])^n^}, whereas K_1/2,D_ is the half-activation concentration of the divalent cation, D, and n is the Hill coefficient.

For dose-response inhibition by NFA, the current (I) measured at the end of the NFA application was normalized to the current amplitude in the absence of NFA (I_0_). The ratio I/I_0_ was then plotted against [NFA], and the concentration-dependent inhibition curve was fitted to a Langmuir function (eq. 2), I/I_0_ = 1/(1+ [NFA]/K_1/2_), where K_1/2_ is the half-inhibition [NFA].

For comparing two treatments that might differentially affect the apparent affinity of the NFA inhibition (for example, Fig. 8 C & D), the summation of the residual error (namely, the residual sum of squares of the error) from the curve fitting of each treatment was compared to the residual error from the curve fitting of the pooled data. If the former was statistically smaller than the latter using F test, the two regression curves would be considered different. The statistically significant level (α) was set at 0.05.

The apparent binding affinity of Mg^2+^ cannot be directly obtained from Mg^2+^ dose-dependent activation curves because Mg^2+^ does not activate ANO1. However, Mg^2+^ appeared to be a competitive inhibitor of Ca^2+^ binding for ANO1 activation, so we constructed a Schild plot, in which the K_1/2,Ca_ in the absence of Mg^2+^ (K_0_) and in the presence of Mg^2+^ (K_Mg_) were compared. In Fig. 4 E, The value of log[(K_Mg_/K_0_)−1] was plotted as a function of [Mg^2+^] (in log scale), and in such a Schild plot the X-axis crossing point of the linear regression line of data points denoted the [Mg^2+^] that increases K_1/2,Ca_ by 2-fold.

The permeability ratios of various anions versus Cl^−^ (P_X_/P_Cl_, where X represents various anions) were calculated using the E_rev_ obtained from bi-ionic experiments according to the following equation (eq. 3), P_X_/P_Cl_ = exp[E_rev_/(RT/F)], where R, T, and F are the gas constant, absolute temperature, and Faraday constant, respectively. Comparing the difference between E_rev_’s of the Ca^2+^- and Sr^2+^-activated current was performed using the pair t test at the significant level of 0.05.

## Supporting Information

Figure S1 **The dead time of observing the effects upon switching solutions was identical for applying Ca^2+^ and NFA.** (A) Comparison between the delay of the Ca^2+^-induced current increase (upper panel) and that of the NFA-induced current reduction (lower panel) in two different patches. Digital signals indicating the start of moving the solution pipes are shown at the bottom of each recording. (B) Averaged delay time for the Ca^2+^ activation and the NFA inhibition. Because this dead time varied from patch to patch, most likely due to different Ω-shape of the patch, we compared the delay of the Ca^2+^-induced current increase and that of the NFA-induced current decrease in the same patch. The difference is not statistically different (pair t-test).
(TIF)

Table S1 **Calculated free [Ca^2+^] in the conditions of 1 mM EGTA, pH = 7.4, 0.14 mM salt solution, temperature = 22°C, with various [Mg^2+^] (in mM).**
(DOC)

Table S2 **Calculated free [Sr^2+^] in the conditions of 0.1 mM EGTA, pH = 7.4, 0.14 mM salt solution, temperature = 22°C.**
(DOC)

Table S3 **Calculated free [Ba^2+^] in the conditions of 0.1 mM EGTA, pH = 7.4, 0.14 mM salt solution, temperature = 22°C.**
(DOC)
