# Supplementary figures and images for: Activation and Inhibition of TMEM16A Calcium-Activated Chloride Channels
Source: PLoS One. 2014 Jan 29;9(1):e86734. doi: 10.1371/journal.pone.0086734 (PMC3906059; doi:10.1371/journal.pone.0086734)

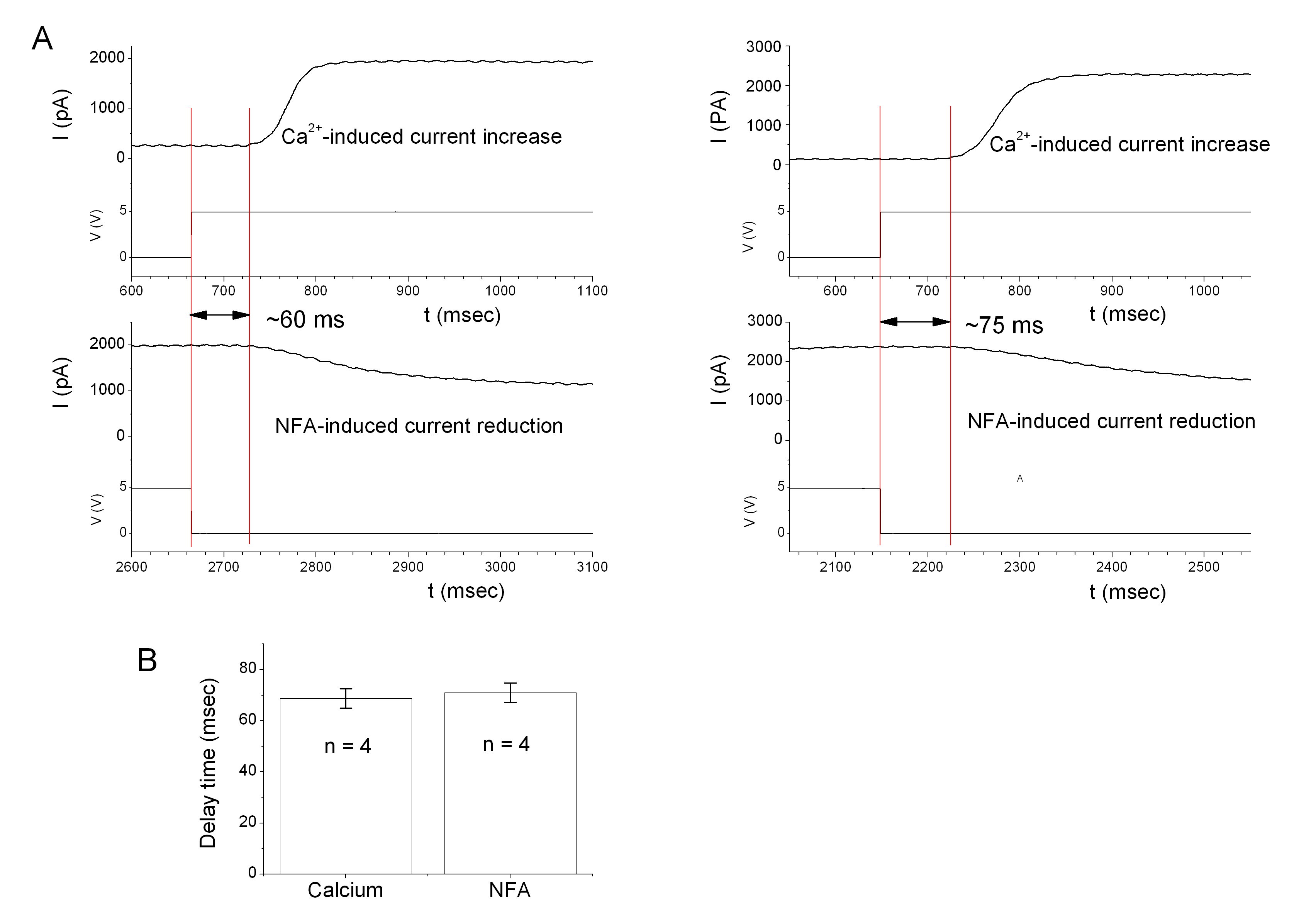

Supplement: Figure S1 — The dead time of observing the effects upon switching solutions was identical for applying Ca2+ and NFA. (A) Comparison between the delay of the Ca2+-induced current increase (upper panel) and that of the NFA-induced current reduction (lower panel) in two different patches. Digital signals indicating the start of moving the solution pipes are shown at the bottom of each recording. (B) Averaged delay time for the Ca2+ activation and the NFA inhibition. Because this dead time varied from patch to patch, most likely due to different Ω-shape of the patch, we compared the delay of the Ca2+-induced current increase and that of the NFA-induced current decrease in the same patch. The difference is not statistically different (pair t-test). (TIF) [file pone.0086734.s001.tif]
