# Supplementary material for: Activation and Inhibition of TMEM16A Calcium-Activated Chloride Channels
Source: PLoS One. 2014 Jan 29;9(1):e86734. doi: 10.1371/journal.pone.0086734 (PMC3906059; doi:10.1371/journal.pone.0086734)
Supplement: Table S1 — Calculated free [Ca2+] in the conditions of 1 mM EGTA, pH = 7.4, 0.14 mM salt solution, temperature = 22°C, with various [Mg2+] (in mM). (DOC) [file pone.0086734.s002.doc]

| Total [Ca^2+^] (mM) | 0.586 | 0.789 | 0.844 | 0.895 | 0.910 | 0.929 | 0.945 | 0.982 |
| --- | --- | --- | --- | --- | --- | --- | --- | --- |
| Free [Ca^2+^] (µM), 0 Mg^2+^ | 0.094 | 0.247 | 0.357 | 0.562 | 0.666 | 0.859 | 1.12 | 3.13 |
| Free [Ca^2+^] (µM), 2 Mg^2+^ | 0.109 | 0.290 | 0.419 | 0.659 | 0.780 | 1.00 | 1.31 | 3.59 |
| Free [Ca^2+^] (µM), 5 Mg^2+^ | 0.134 | 0.354 | 0.512 | 0.803 | 0.951 | 1.22 | 1.59 | 4.25 |
| Free [Ca^2+^] (µM), 10 Mg^2+^ | 0.175 | 0.461 | 0.666 | 1.04 | -- | 1.58 | 2.05 | 5.28 |
