# Supplementary material for: Activation and Inhibition of TMEM16A Calcium-Activated Chloride Channels
Source: PLoS One. 2014 Jan 29;9(1):e86734. doi: 10.1371/journal.pone.0086734 (PMC3906059; doi:10.1371/journal.pone.0086734)
Supplement: Table S2 — Calculated free [Sr2+] in the conditions of 0.1 mM EGTA, pH = 7.4, 0.14 mM salt solution, temperature = 22°C. (DOC) [file pone.0086734.s003.doc]

| Total [Sr^2+^] (µM) | 10 | 20 | 50 | 100 | 200 | 500 |
| --- | --- | --- | --- | --- | --- | --- |
| Free [Sr^2+^] (µM) | 1.7 | 3.67 | 11.6 | 35.0 | 114 | 404 |
