# Supplementary material for: Activation and Inhibition of TMEM16A Calcium-Activated Chloride Channels
Source: PLoS One. 2014 Jan 29;9(1):e86734. doi: 10.1371/journal.pone.0086734 (PMC3906059; doi:10.1371/journal.pone.0086734)
Supplement: Table S3 — Calculated free [Ba2+] in the conditions of 0.1 mM EGTA, pH = 7.4, 0.14 mM salt solution, temperature = 22°C. (DOC) [file pone.0086734.s004.doc]

| Total [Ba^2+^] (mM) | 0.05 | 0.1 | 0.2 | 0.5 | 2 | 5 |
| --- | --- | --- | --- | --- | --- | --- |
| Free [Ba^2+^] (µM) | 13.5 | 38.2 | 116 | 405 | 1900 | 4900 |
